# A Study Protocol: Engagement of Lived Experience Voices for Analysis of Transformative Evidence in Mental Health Policy & Legislation (ELEVATE-MH)

**DOI:** 10.1371/journal.pone.0346037

**Published:** 2026-04-15

**Authors:** Kalo C. Sokoto, Tina W. Masai, Sisikelelwe S. Gwanya-Mdletye, Olindah Silaule, S’thembile Phindile Promise Jili, Selamawit A. Tessema, Mohhadiah Rafique, Ghonda Lepkeh, Action Amos, Kimberly Kariuki, Yiknashewa Solomon, Senait Ghebrehiwet, Sarah E Valentine, David C. Henderson, Benjamin Harris, Chiliza Bonginkosi, Martyn Pickersgill, Lucinda Manda-Taylor

**Affiliations:** 1 Department of Psychiatry, Boston Medical Center, Boston, Massachusetts, United States of America; 2 Kasa Mind and Wellness Institute, Nairobi, Kenya; 3 Walter Sisulu University, Eastern Cape Province, South Africa; 4 Department of Health and Rehabilitation Sciences, Division of Occupational Therapy, University of Cape Town, South Africa; 5 Department of Psychiatry, University of KwaZulu-Natal, Durban, South Africa; 6 Department of Psychiatry, St. Paul’s Hospital Millennium Medical College, Addis Ababa, Ethiopia; 7 Department of Psychiatry at Stellenbosch University, South Africa; 8 Department of Psychiatry, University of Liberia, Monrovia, Liberia; 9 International Bureau of Epilepsy, Blantyre, Malawi; 10 Centre for Clinical Brain Science, College of Medicine & Veterinary Medicine, The University of Edinburgh, Scotland; 11 Non-communicable Diseases Research Programme, Kenya Medical Research Institute, Kenya; 12 Department of Psychiatry, St. Peter’s Specialized Hospital, Ethiopia; 13 Centre for Biomedicine, Self and Society, School of Population Health Sciences, The University of Edinburgh, United Kingdom; 14 Training and Research Unit of Excellence, Blantyre, Malawi; 15 Kamuzu University of Health Sciences, Blantyre, Malawi; Public Library of Science, UNITED KINGDOM OF GREAT BRITAIN AND NORTHERN IRELAND

## Abstract

Mental health conditions affect almost one in seven people globally. In response to the Convention on the Rights of Persons with Disabilities (CRPD), efforts to strengthen mental health systems have included increased funding, destigmatization of mental health, and the engagement of People with Lived Experience (PWLE) in decision making. Yet the extent to which PWLE are engaged in mental health policy development is not well characterized in the literature. To that end, this study seeks to understand the engagement of PWLE in mental health policy making in Africa and identify strategies for enhancing meaningful engagement of PWLE. The study described in this protocol will employ a qualitative multi-methods approach, utilizing the Policy Triangle Framework to analyze the policy making process, and International Association for Public Participation Spectrum to identify the extent of PWLE engagement. We will perform a scoping review of national mental health policies and legislation. We will interview 50 key informants including mental health policymakers, advocates, and researchers, and conduct focus group discussions with 120 PWLE. Qualitative data will be subject to content and thematic analysis. Country-specific analyses will be followed by a cross-country synthesis. Findings will inform context specific and strategies to enhance meaningful engagement of PWLE in mental health policy making. These will be disseminated through stakeholder workshops in each country, academic publications, conferences, PWLE-led knowledge translation activities, and policy briefs co-authored with community advocates.

## Introduction

Mental health conditions are a leading cause of disability globally with nearly one in seven people living with a mental disorder [1]. Evidence-based mental health policies and legislation are system-level strategies aimed at prevention and treatment of mental health concerns [2]. In places where mental health systems and evidence infrastructure are still developing, foreign and empirical research evidence has predominantly informed mental health policies [3,4]. Across Low and Middle Income Countries (LMIC’s), social evidence, which refers to the insights and experiences of individuals and communities affected by mental health issues, has received significantly less attention in mental health policy making [5,6].

Following the adoption of the Convention on the Rights of Persons with Disabilities (CRPD), there has been an increased interest in engaging People With Lived Experience (PWLE’s) in policy making [7,8]. Engaging PWLE has been described as “a person-centered approach, with bidirectional information flow, power sharing and access to advocacy at a personal, service and/or societal level” [9]. Benefits of engaging PWLE in policy making include improved understanding of the challenges faced by PWLE, more responsive policies and programs, effective use of resources, increased trust between PWLE and health establishments, increased mental health service utilization, and an increased satisfaction with mental health services [10–12]. Despite these documented benefits, the extent to which PWLE are engaged in mental health policymaking remains limited.

PWLE are the least engaged stakeholders in mental health policy making [12,13]. The exclusion of PWLE occurs across all levels of policy making including planning, implementation and evaluation [14,15]. Identified barriers to including PWLE in policy making include stigma, sexism, and lack of standardized practices [16]. Factors that enable entities (i.e., government leaders, institutions, and programs) include a focus on human rights and established mechanisms for engagement of PWLE [17]. To date, there is a dearth of studies evaluating the engagement of PWLE in mental health policy development in LMIC’s [18–20]. Identifying practices for engaging PWLE in mental health policy making in these countries may promote the development of context-specific, evidence-informed policies that uphold human rights.

There is an urgent need to reimagine mental health policymaking in ways that are inclusive, contextually relevant, and grounded in evidence [21,5]. Responsive and equitable mental health systems require the engagement of persons with lived experience as a form of evidence that can support rights-based and participatory policy development [22,23]. The Engagement of Lived Experience Voices for Analysis of Transformative Evidence in Mental Health Policy & Legislation (ELEVATE-MH) study described in this protocol aims to contribute to the evolving discourse on meaningfully engaging PWLE in the strengthening of mental health systems including policies and legislature. ELEVATE–MH is a British Academy funded study across 2025–2026.

The ELEVATE-MH study described in this protocol aims to examine the extent, nature, and implications of engagement of People With Lived Experience (PWLE) in mental health policy-making across Liberia, Malawi, Ethiopia, Kenya, and South Africa. Our specific objectives are to:

1. To map and analyse the extent to which PWLE are engaged in national mental health policy and legislation.
2. Examine how PWLE are engaged in the policy and legislation making process.
3. Identify facilitators and barriers of engaging with PLWE evidence in mental health policy making.
4. Identify context-specific strategies to enhance engagement of PWLE evidence in mental health policymaking.

## Materials and methods

**Fig 1:**
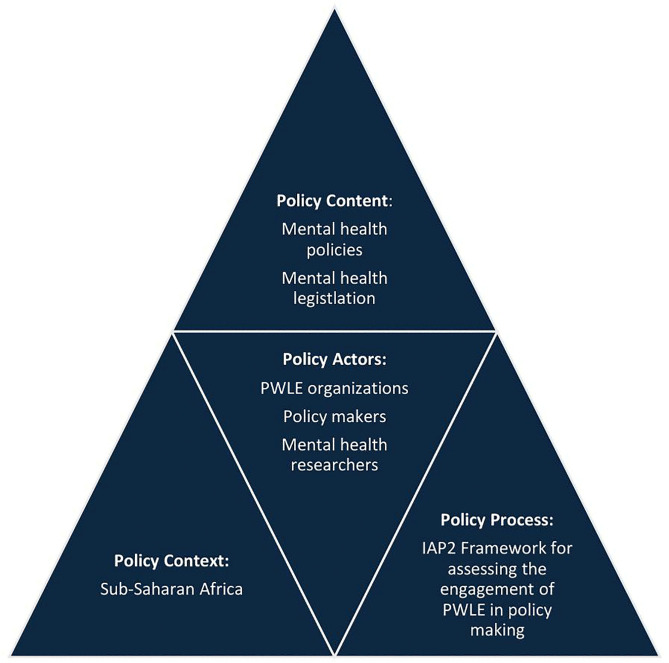
Policy Triangle Framework applied to the ELEVATE-MH study.

The development of our protocol was guided by the Policy Triangle Framework [24]. This examines health policy through the interconnected dimensions of content, context, process, and actors, while also considering the role of power. It focuses on the different elements involved in policy making and can therefore place emphasis on who is involved in policy making. (See Fig 1)

### Study design

This study will employ a qualitative multi-methods design. This will include a desk review and a qualitative analysis. To address Objective 1 a scoping review using standardized extraction tool will be conducted to map and analyze the characteristics of national mental health policies and legislation in Africa. This will include an assessment of policy priorities, legal frameworks, references to social data, and the documented inclusion of PWLE. The findings from this review will also contribute to identifying and quantifying the extent to which integration of social data into mental health policymaking across these countries.

To complement the document analysis and address objectives 2, 3 and 4, qualitative data will be gathered through focus group discussions with PWLE will be conducted to examine collective perspectives on the extent, nature, and experience of PWLE engagement in mental health policy making. Additionally, in-depth interviews with key informants in the selected countries, including policymakers, mental health professionals, and researchers to understand the value, relevance, and influence of social data, especially those generated by PWLE, in shaping mental health policy. This design enables the triangulation of findings across multiple data sources, countries and perspectives, thereby enhancing the credibility and utility of the results. It is particularly well-suited to exploring the nuanced and context-specific dynamics of policy development and implementation, including the engagement of PWLE in mental health policymaking processes across diverse African settings.

### Study setting

The countries where this study will be conducted include Liberia, Malawi, Ethiopia, Kenya, and South Africa. Considerations that informed the choice of counties included availability of mental health policies and legislations, regional diversity, and access to in-country partners. A spreadsheet/Google Sheet form was created with all 55 African countries. Mental health policies and legislations for African countries were identified using the World Mental Health Atlas 2024 [25]. We categorized countries with both mental health policy and legislations into regions on the African continent which included North, West, South, South West and East Africa. Finally, we considered which countries in each region members of the research team had access to in-country stakeholders. The rationale being that an established relationship ensured that this study will be able to navigate the in-country systems more effectively than if we had no established relationships. These countries also represent a range of political and economic contexts which will inform our study. Within each country, data will be collected at the national level, focusing on mental health policymaking bodies, relevant ministries, non-governmental organizations and PWLE-led advocacy networks.

### Study participants

The study participants for this study will include three key informant groups across the selected countries:

1. PWLE of mental health conditions, namely mental health service users and families/caregivers.
2. Policymakers involved in health policy development and implementation, including individuals working within government ministries, departments, or statutory bodies.
3. Academic, independent researchers or advocates who inform policy processes

The eligibility criteria for this study include participants aged 18–65 years, self-identifying as belonging to one of the three key informant groups, being a resident or national of one of the five study countries, and providing informed consent to audio recording.

### Study period

**Fig 2:**
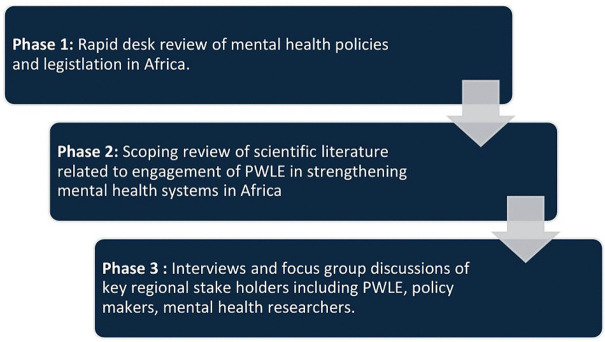
ELEVATE-MH study phases.

This study will be conducted across 2025–2026. Currently, ethics approvals have been obtained for all participating countries. The first phase began in May 2025 and estimated to complete in May 2026. The second phase began in October 2025 and estimated complete in May 2026. The third phase began in January 2026. Specifically, participant recruitment began on 17^th^ January 2026 in Ethiopia, 19^th^ January 2026 in Malawi, 23^rd^ February 2026 in Kenya, and February 12^th^ 2026 in Liberia. South Africa anticipates to start participant recruitment in April 2026. Participant recruitment is anticipated to be completed by May 2026 and data collection by July 2026. Data analysis and cross-country synthesis will take place between July and October 2026, with preliminary results anticipated in November 2026. The study will be conducted in three phases (See Fig 2).

### Phase One: Mapping and Analyzing Mental Health Policies and Legislations in Africa

The first phase will seek to answer the following questions:

1. What regional policies and legislations exist to tackle mental health challenges in Africa?
2. Who are the key stakeholders (i.e., policymakers, service users, caregivers, and healthcare workers) in the mental health policymaking process?
3. What role do PWLE play in mental health policymaking?

## Methodology

Mental health policies and legislations for African countries will be identified through the World Mental Health Atlas 2024 platform. Using a validated extraction tool we will identify information from the policy documents that will answer the earlier stated questions [26] (S1 Table).

### Analysis

After this information is collected, three independent researchers will discuss findings and reach consensus on (1) mental health policies and legislations in Africa (2) key stake holders in mental health policymaking, and 3) engagement of PWLE in policy making.

### Phase Two: Scoping Review of Scientific Literature Related to Engagement of PWLE in Strengthening Mental Health Systems in Sub-Saharan Africa

The second phase of the project will aim to conduct a scoping review guided by Arkesey and O’Malley’s methodological framework [27]. This approach involves five key steps: (1) identifying the research question, (2) identifying relevant studies and policy documents, (3) selecting studies based on predefined inclusion and exclusion criteria, (4) charting the data using a structured extraction framework, and (5) collating, summarizing, and reporting the results to highlight key themes, gaps, and implications for policy and practice.The scoping review aims to answer the following questions in addition to expanding the questions in phase one.

1. What roles do PWLE play in mental health policymaking processes?
2. What are the barriers and facilitators influencing the engagement of PWLE evidence in mental health policymaking across Sub-Saharan Africa?
3. What are the context-specific strategies for the engagement of PWLE evidence in policy making?

### Search strategy

A three-step search strategy process will be used to identify relevant studies related to engagement of PWLE in mental health policy making. An initial scoping of the literature will be conducted using a focused set of keywords on PubMed, MEDLINE, PsycINFO, Scopus, Web of Science and African Journals Online (AJOL), Google Scholar and Grey literature sources. The initial keywords will include “mental health policy OR mental health legislation OR mental health framework OR mental health strategy OR mental health guideline” AND “ LMICs OR developing country OR sub-Saharan Africa” AND “Policymakers AND mental health service users (PWLE) AND families OR caregivers AND mental healthcare workers” AND “Evidence-based OR Evidence-Informed OR Evidence” AND “ Engagement OR inclusion OR diverse, engagement of People with Lived Experience, engaging PWLE, Care giver involvement, User involvement, Patient involvement, Co-production, Co-design, user-focused. ” The second search will include all keywords and index terms identified and revised with the assistance of a librarian at the University of Kwa-Zulu-Natal. Lastly, the reference lists of all included sources of evidence will be screened for additional studies. Only studies published in English will be considered for inclusion.

### Inclusion criteria

The review will be limited to peer-reviewed articles and literature between the years 2000 and 2025. Literature published within the last two decades, Africa-specific policy recommendations for mental health, empirical, conceptual, and grey literature on evidence-informed mental health policy making, mental health policy development, formulation implementation, or evaluation in Sub-Saharan African countries, populations affected by mental health policies in Sub-Saharan Africa including service users, healthcare workers, and policy makers, and key literature identified as foundational or widely cited.

Articles published more than 25 years ago will be excluded, except when a country’s only available mental health policy originates from the colonial period. Additional exclusion criteria include research not focused on a country or countries within the African continent. Publications that do not engage with mental health policymaking and studies that do not address any stage of policy development or implementation.

### Screening

The identified sources will be uploaded to Covidence, and any duplicates will be removed before screening. Initial screening will entail the screening of titles and abstracts for eligibility based on the above inclusion and exclusion criteria. For those which meet criteria, full articles will be retrieved and screened to confirm eligibility. The papers which meet criteria shall be shared between two reviewers who will review the articles against a data charting framework and document their findings on Covidence. Papers reviewed by the first and second reviewer will be reviewed by third and fourth reviewers. Differences will be discussed between the four reviewers until consensus is reached.

### Analysis

Each reviewer will apply content analysis to the selected papers. Reviewers will come together to discuss any inconsistencies and work towards a consensus. After consensus, the reviewers will summarize patterns relating to each research question. This will be the basis of their narrative synthesis.

### Phase Three: Interviews and Focus Group Discussions of Key Regional Stakeholders Including PWLE, Policy Makers, Mental Health Researchers and Mental Health Advocates

**Fig 3:**
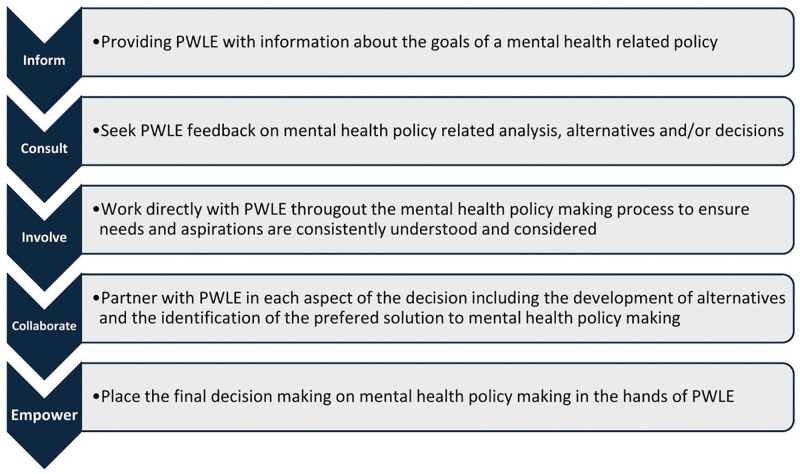
International Association for Public Participation (IAP2) Spectrum Framework applied to the engagement of People With Lived Experience (PWLE) in mental health policy making.

The final phase of the project will entail qualitative interviews with key informants in the five study countries. The interview and focus group discussion guides will be informed by The International Association for Public Participation Spectrum (IAP2) [28]. The IAP2 Framework is a widely used framework for categorizing levels of stakeholder engagement in decision-making processes. It identifies five levels of engagement: Inform, Consult, Involve, Collaborate, and Empower, each reflecting an increasing degree of public influence and power-sharing. The IAP2 Spectrum can guide the assessment of how PWLE are engaged in mental health policy development, implementation, and evaluation, and help identify opportunities to strengthen meaningful and equitable participation. (See Fig 3)

Interview and focus group discussion guides were informed by a literature review, input from clinicians, and key informants that included people with lived experience. Examples of questions from the interview guide include:1) Have you (or your organization) ever been asked to provide input into a mental health policy or program?2) If yes: What was the experience like for you?, and 3)What can be done to better engage people with lived experience in mental health policy making? The interview and focus group discussion guides will be translated into national languages of all five countries and pilot-tested with 2–3 participants prior to data collections. The guides allows flexibility for interviewers to follow participant narratives while ensuring consistency across interviews (S1 File & S2 File). A demographics survey will also be administered to all participants (S3 File).

The project team will conduct in-depth interviews with policymakers, researchers, and PWLE’s and focus group discussions with PWLE. The total number of participants we aim to recruit is n = 170. From each of the five countries, the study aims to recruit 5 mental health researchers, 5 mental health policy makers or advocates, and 24 PWLE’s. The aim of the qualitative data collection is to gain deeper insights into PWLE engagement in the policy development process. The total number of individual interviews (n = 50) and focus groups (n = 15) is informed by established recommendations to achieve saturation of data in qualitative studies for homogenous groups with clear study objectives [29].

### Sampling

Participants will be recruited via national PWLE organizations, government departments, and mental health non-governmental organizations. A snowball sampling strategy will be employed, beginning with established organizations that collaborate with PWLE and mental health advocacy groups. All eligible research participants will be approached individually, either via email or in person, depending on the context, with an invitation to participate. Written informed consent will be obtained. The consent process will be conducted in English and the local language (e.g., Amharic, Chichewa, Kiswahili, or isiZulu) by trained research assistants. Each country site will train their research assistants.

Data collection will take place both online via Zoom or in person, depending on the setting. Reasonable accommodations such as flexible scheduling for data collection, break provisions, and extended time-frames where required will be integrated to ensure meaningful PWLE engagement during data collection, analysis and results dissemination.

### Analysis

Interviews will be transcribed verbatim by two researchers from each country and managed using NVivo 14. When interviews are conducted in local languages, transcripts will be translated and back translated. An in-country team-based coding process will be applied including familiarization, categorization, identifying, reviewing and refining themes. In-country coding frameworks will be developed collaboratively. Analysis will follow thematic analysis, using an deductive approach. Country-specific analyses will then be conducted first, to account for contextual differences, followed by a cross-country synthesis to identify common themes and contrasts. Two additional researchers will lead the cross country analysis.

### Data management

The data that will be obtained from and/or about study participants and potential participants will be the minimum necessary to conduct the study. All study data and documents will be recorded as anonymous. There will be no master code, and no reasonable way to link study data and documents to individual participants. Participant contact information will be maintained separately in a secure server for administrative purposes (e.g., participant enrollment and compensation) and will not be associated with any personal data. Only the in-country research team will have access to in-country contact information.

All qualitative interviews and focus groups will be recorded to ensure accurate transcription. When sessions are conducted via Zoom, the HIPAA-compliant Zoom transcription feature will be used. For in-person sessions, handheld digital recorders will be used. In addition, trained research assistants will take detailed field notes during each session. Recordings from handheld devices and field notes will be deidentified and uploaded to a secure, access-controlled in-country institution server within 24 hours of data collection.

All research data will be stored in compliance with the in-country institutions’ policies for secure storage of data containing Protected Health Information (PHI) and/or Personal Information, in accordance with in-country legislation. Study data pertaining to a given country will be securely stored on each institution’s protected platform within their respective country for 5 years following study completion.

### Ethical consideration

Ethics approval was sought from institutions within the five countries. The University of Liberia in Liberia, Kamuzu University of Health Sciences College of Medicine Research Committee (COMREC) in Malawi, St Paul’s Hospital Millennium Medical College Institutional Research Ethics Review Committee in Ethiopia, The Mount Kenya University Institutional Scientific and Ethical Review Committee (ISERC) in Kenya, and University of Kwazulu-Natal’s Humanities and Social Sciences Research Ethics Committee (HSSREC) in South Africa.

## Discussion

### Strengths and limitations

This study will highlight the extent of engagement of PWLE in mental health policy making in Africa, identify the facilitators and barriers of engaging PWLE evidence in policymaking, and in so doing make recommendations regarding context specific strategies of engaging PWLE evidence in mental health policymaking. In so doing, this study will contribute to enhancing engagement of PWLE in mental health policymaking as one way to strengthen mental health systems. This is a significant contribution given current global efforts to meaningfully engage PWLE in policy making.

While this is a multi-country study with the goal of obtaining regional perspectives across Africa, generalizability of qualitative findings beyond the specific contexts examined is a key limitation.

### Dissemination plans

Results from the study will be shared through a variety of mechanisms including publishing, conferences, stakeholder workshops and PWLE-led knowledge translation activities. The study aims to publish a scoping review and cross country study findings. The purpose of these publications is to synthesize literature on engagement of PWLE in policy making in Africa, and document strategies of engaging PWLE in mental health policy making in Africa. The intended outcome is, ultimately, for an increase in engagement of PWLE in policy making.

To achieve this, the research team will carry out PWLE-led community forums and advocacy meetings to ensure impact. We aim to present findings to PWLE organizations across the participant countries. We also aim to have findings tabled in discussion sessions with researchers and policymakers, and provide capacity building workshops in engaging PWLE in policy making. We will collaboratively advocate to national governments ministries of health that meaningful engagement of PWLE in health policy will be a requirement by national governments.

### Risk-reducing steps

Considering the documented harms that have been caused in engaging PWLE in research, the greatest risk to the success of this project is in perpetuating these harms. Some of these harms include emotional or psychological harm through recollection of painful experiences, lack of clarity in the informed consent process, confidentiality of data, power imbalances between researchers and participants, stigmatization, lack of agency, and misinterpretation of findings.

To address these potential risks, the research team is engaging established best practices in engaging PWLE in research. For one, persons with lived experience are engaged throughout the research process ensuring co-production. Collaborating with PWLE organizations across the five countries will be the source of recruitment of participants for the study. Further, compensation for PWLE engagement has been allocated, compliance to ethics approval, researcher’s ethical reflexivity and commitment to provide capacity building to participants are additional steps taken by the research team to ensure responsible engagement with PWLE.

## Supporting information

S1 Table Policy Data Extraction Tool. Data extraction table for policy documents adapted from Peters et al. (2020).
(DOCX)

S1 File Focus Group Discussion Guide. Semi-structured focus group discussion guide for People With Lived Experience (PWLE).
(DOCX)

S2 File Key Informant Interview Guide. Semi-structured in-depth interview guide for policymakers, researchers, and advisors.
(DOCX)

S3 File Demographics Questionnaire. Participant demographics questionnaire administered to all study participants.
(DOCX)

S4 File PRISMA-P Checklist File. Completed PRISMA-P (Preferred Reporting Items for Systematic Review and Meta-Analysis Protocols) 2020 checklist for the Phase 2 scoping review component of the ELEVATE-MH study.
(DOCX)
