# Supplementary material for: A Study Protocol: Engagement of Lived Experience Voices for Analysis of Transformative Evidence in Mental Health Policy & Legislation (ELEVATE-MH)
Source: PLoS One. 2026 Apr 15;21(4):e0346037. doi: 10.1371/journal.pone.0346037 (PMC13082620; doi:10.1371/journal.pone.0346037)
Supplement: S1 Table — Data extraction table for policy documents adapted from Peters et al. (2020). (DOCX) [file pone.0346037.s001.docx]

**Appendix 1:** Data extraction table for policy documents adapted from Peters et.al. (2020)

| **Scoping Review Details:** | | |
| --- | --- | --- |
| Scoping Review Title |  | |
| Scoping Review Objective/s |  | |
| Scoping Review question/s |  | |
| **Inclusion/Exclusion Criteria:** | | |
| Population |  | |
| Concept |  | |
| Context | Country | |
|  | Type of setting(urban/peri-urban/rural) | |
| Type/s of evidence source |  | |
| **Evidence source Details and Characteristics:** | | |
| Aim/s of the policy document |  | |
| Objective/s of the policy document |  | |
| Citation details | Author/s | |
|  | Date | |
|  | Title | |
|  | Pages | |
| Policy Actors Involved |  | |
| Country |  | |
| Context(urban/peri-urban/rural) |  | |
| Target population | Age | |
|  | Gender | |
| Characteristics of policy document |  |  |
|  | Evidence-Informedness | |
|  | Execution plan | |
|  | Defined procedure | |
|  | Periodical review | |
| Stage of Policy Addressed |  | |
| Targeted outputs |  | |
| **Details extracted from source of evidence:** | | |
| Overview of details |  | |
| Gaps Identified |  | |
| **Recommendations extracted from source of evidence:** | | |
| Conclusions |  | |
| Recommendations |  | |
