# Supplementary material for: A Study Protocol: Engagement of Lived Experience Voices for Analysis of Transformative Evidence in Mental Health Policy & Legislation (ELEVATE-MH)
Source: PLoS One. 2026 Apr 15;21(4):e0346037. doi: 10.1371/journal.pone.0346037 (PMC13082620; doi:10.1371/journal.pone.0346037)
Supplement: S1 File — Semi-structured focus group discussion guide for People With Lived Experience (PWLE). (DOCX) [file pone.0346037.s002.docx]

**Appendix 3: Focus Group Discussion Guide – People With Lived Experience (PWLE)**

**Title of the Study:** *Engagement of Lived Experience Voices for Analysis of Transformative Evidence in Mental Health Policy & Legislation (ELEVATE-MH)*

**Facilitator Introduction**
Thank you all for agreeing to participate in this focus group discussion. My name is __________________, and I am part of a research team studying how people with lived experience of mental health conditions are engaged in mental health policy-making across several African countries. We are independent researchers and do not work for the government.

We are interested in hearing your thoughts, experiences, and perspectives. There are no right or wrong answers. We want you to feel comfortable sharing your views at your own pace. The discussion will take about 60–90 minutes.

Participation is voluntary, and everything you share will be kept confidential. Please respect each other's privacy and avoid sharing personal stories outside this group.
Before we begin, does anyone have any questions?

**A. Participant Demographic Information (Round-Robin on a Register)**

Please introduce yourself with the following (only what you feel comfortable sharing):

- Age
- Sex
- Marital status
- Highest level of education
- Occupation/livelihood

**B. Introduction and Rapport Building**

- To start, please tell us a little about yourselves.
  *Probe:* What made you interested in participating in this discussion?
- Can you tell us about your experience living with, or supporting someone with, a mental health condition?
- Have you ever interacted with policymakers or government officials about mental health issues?
  *Probe:* In what context? How did that interaction happen?

**C. Perceptions of Mental Health Advocacy**

- Have you been involved in mental health advocacy or policy activities?
  *Probe:* If yes, what were you involved in? How did you participate?
- Has you or your organisation ever been asked to provide input into a mental health policy or programme?
  *Prompts:*
  - Who else was involved?
  - How were you contacted or selected?
  - How did you learn about the opportunity?
  - How were these opportunities communicated to your community?
  - What parts of the policy reflected your lived reality, and what was missing?
- From your perspective, what issues are missing from current mental health policies?
  *Probe:* What do you think should be added to make policies more responsive?

**D. Role and Inclusion in Policymaking (Process)**

- Are there examples where your voice—or the voices of other PWLE—helped shape a policy, service, or outcome?
- What roles have you played when you were invited to participate in policy processes?
  *Probes:*
  - Sharing experiences
  - Giving feedback
  - Helping design or monitor programmes
  - Did you feel listened to? Were your suggestions taken seriously?
- To what extent did you feel involved in the process?
  *Prompt:* Just consulted? Involved? Collaborating? Empowered?
- At what stage were you brought into the process?
  *Probe:* Early in policy development or after major decisions were already made?
- Did you receive feedback after you provided your input?
  *Probes:*
  - Were you informed of decisions made?
  - Did you see results?

**E. Barriers and Enablers (Context)**

1. Based on your experience, what are the main barriers to PWLEs being meaningfully involved in policymaking?
2. Are there people, organisations, groups, or spaces that have helped elevate PWLE voices?
   *Probe:* What made these spaces supportive?
3. Have you experienced stigma or discrimination while engaging with policymakers or other stakeholders?
   *Probe:* If so, in what situations?

**F. Recommendations (IAP2 Framework)**

1. How can information about mental health policies be communicated better to PWLE? *(Inform)*
2. What is the best way for policymakers to consult PWLE about policies or services? *(Consult)*
3. How can PWLE be more meaningfully involved throughout the policy cycle—not only during consultations? *(Involve/Collaborate)*
4. What would true collaboration with policymakers look like for PWLE? *(Collaborate)*
5. What would empowerment mean to you in mental health policymaking?
   *Prompt:* Having influence, shared decision-making power, and co-leadership.
6. What support would make your participation in policy-making easier?
   *Probes:* transportation, training, compensation, and accessible meeting spaces.

**Closing**

Thank you all for participating and sharing your experiences and ideas. Your perspectives are essential to improving mental health policies and ensuring that lived experience voices are meaningfully included.
Before we finish, is there anything else anyone would like to add?
