# Supplementary material for: A Study Protocol: Engagement of Lived Experience Voices for Analysis of Transformative Evidence in Mental Health Policy & Legislation (ELEVATE-MH)
Source: PLoS One. 2026 Apr 15;21(4):e0346037. doi: 10.1371/journal.pone.0346037 (PMC13082620; doi:10.1371/journal.pone.0346037)
Supplement: S2 File — Semi-structured in-depth interview guide for policymakers, researchers, and advisors. (DOCX) [file pone.0346037.s003.docx]

**Appendix 2: Interview Guide - Policymakers, Researchers, and Advisors**

**Title of the Study:** *Engagement of Lived Experience Voices for Analysis of Transformative Evidence in Mental Health Policy & Legislation (ELEVATE-MH)*

**Facilitator Introduction**
Thank you all for agreeing to participate in this In-depth interview. My name is __________________, and I am part of a research team studying how people with lived experience of mental health conditions are engaged in mental health policy-making across several African countries. We are independent researchers and do not work for the government.

We are interested in hearing your thoughts, experiences, and perspectives. There are no right or wrong answers. We want you to feel comfortable sharing your views at your own pace. The Interview will take about 60–90 minutes.

Participation is voluntary, and everything you share will be kept confidential. Please respect each other's privacy and avoid sharing personal stories outside this group.
Before we begin, does anyone have any questions?

1. **Background and Role**

- Can you tell me about your role and experiences around mental health policy making?
- How long have you been in the role/expertise around mental health policy making?
- Can you describe your role in mental health policy or program development?
- What is the policy/program development process in your institution/ministry? How are the process of decision making about mental health made in your institution or ministry?

1. **Evidence Use and Policy Content**

- What types of evidence do you rely on when developing or evaluating mental health policy?
- In your role as a policymaker/researcher, what informs your use of experiential data (e.g. input from PWLE, families or caregivers?)
- Are there any examples where lived experience influenced a policy or program?

1. **Policy Process and Inclusion (Mapped to IAP2 Spectrum)**

- Do stakeholders (including PWLE) tend to be identified and included during the policy process? (**Consult/Involve**)
  - If so, how?
- In what ways are PWLE informed about policies or decisions that may affect them? (**Inform**)
- How are PWLE consulted (surveys, forums, working groups)? At what points in policymaking/research are they consulted? (Probe on frequency, role: co-developing or coleading (**Consult/collaborate**)
- Are there examples where PWLE were part of **co-developing** policies or co-leading initiatives? (**Collaborate**)
- Have you encountered cases where PWLE were given shared decision-making power or authority? (**Empower**)
- At what stages (development, implementation, monitoring) are PWLE typically engaged?
- How do you ensure that PWLE input influences the final outcomes?

1. **Policy Context and Enablers/Barriers**

- What are the biggest challenges to using experiential data in policy processes?
- In your opinion, what helps/enables PWLE participation?
- Conversely, what hinders PWLE participation (e.g., political will, funding, public stigma)?
- How does stigma and discrimination affect whether and how PWLE are included?

1. **Reflection and Recommendations (Framed by IAP2)**

- What would improve the **transparency and communication** of mental health policy to affected communities? (**Inform**)
- What are effective methods for **consulting** communities and PWLE, especially those most marginalized? (**Consult**)
- Are there any ways that you organization could **involve** PWLE more meaningfully and consistently in the policy cycle that – in an ideal world – you would like to try? (**Involve**)
- What recommendations do you have for policy makers who would like to implement the involvement of PWLE in policymaking? (**Collaborate**)
- Are there examples—locally or internationally—where PWLE have been **empowered** to lead or co-own decision-making? (**Empower**)
- Going back to the notion of an ideal world, if money, resources, and time were no issue, how would you like mental health policy to develop?

**Closing**

- Thank you all for participating and sharing your experiences and ideas. Your perspectives are essential to improving mental health policies and ensuring that lived experience voices are meaningfully included.
  Before we finish, is there anything else anyone would like to add?

**Appendix 3: Focus Group Discussion Guide – People With Lived Experience (PWLE)**

**Title of the Study:** *Engagement of Lived Experience Voices for Analysis of Transformative Evidence in Mental Health Policy & Legislation (ELEVATE-MH)*

**Facilitator Introduction**
Thank you all for agreeing to participate in this focus group discussion. My name is __________________, and I am part of a research team studying how people with lived experience of mental health conditions are engaged in mental health policy-making across several African countries. We are independent researchers and do not work for the government.

We are interested in hearing your thoughts, experiences, and perspectives. There are no right or wrong answers. We want you to feel comfortable sharing your views at your own pace. The discussion will take about 60–90 minutes.

Participation is voluntary, and everything you share will be kept confidential. Please respect each other's privacy and avoid sharing personal stories outside this group.
Before we begin, does anyone have any questions?

**A. Participant Demographic Information (Round-Robin on a Register)**

Please introduce yourself with the following (only what you feel comfortable sharing):

- Age
- Sex
- Marital status
- Highest level of education
- Occupation/livelihood

**B. Introduction and Rapport Building**

- To start, please tell us a little about yourselves.
  *Probe:* What made you interested in participating in this discussion?
- Can you tell us about your experience living with, or supporting someone with, a mental health condition?
- Have you ever interacted with policymakers or government officials about mental health issues?
  *Probe:* In what context? How did that interaction happen?

**C. Perceptions of Mental Health Advocacy**

- Have you been involved in mental health advocacy or policy activities?
  *Probe:* If yes, what were you involved in? How did you participate?
- Has you or your organisation ever been asked to provide input into a mental health policy or programme?
  *Prompts:*
  - Who else was involved?
  - How were you contacted or selected?
  - How did you learn about the opportunity?
  - How were these opportunities communicated to your community?
  - What parts of the policy reflected your lived reality, and what was missing?
- From your perspective, what issues are missing from current mental health policies?
  *Probe:* What do you think should be added to make policies more responsive?

**D. Role and Inclusion in Policymaking (Process)**

- Are there examples where your voice—or the voices of other PWLE—helped shape a policy, service, or outcome?
- What roles have you played when you were invited to participate in policy processes?
  *Probes:*
  - Sharing experiences
  - Giving feedback
  - Helping design or monitor programmes
  - Did you feel listened to? Were your suggestions taken seriously?
- To what extent did you feel involved in the process?
  *Prompt:* Just consulted? Involved? Collaborating? Empowered?
- At what stage were you brought into the process?
  *Probe:* Early in policy development or after major decisions were already made?
- Did you receive feedback after you provided your input?
  *Probes:*
  - Were you informed of decisions made?
  - Did you see results?

**E. Barriers and Enablers (Context)**

1. Based on your experience, what are the main barriers to PWLEs being meaningfully involved in policymaking?
2. Are there people, organisations, groups, or spaces that have helped elevate PWLE voices?
   *Probe:* What made these spaces supportive?
3. Have you experienced stigma or discrimination while engaging with policymakers or other stakeholders?
   *Probe:* If so, in what situations?

**F. Recommendations (IAP2 Framework)**

1. How can information about mental health policies be communicated better to PWLE? *(Inform)*
2. What is the best way for policymakers to consult PWLE about policies or services? *(Consult)*
3. How can PWLE be more meaningfully involved throughout the policy cycle—not only during consultations? *(Involve/Collaborate)*
4. What would true collaboration with policymakers look like for PWLE? *(Collaborate)*
5. What would empowerment mean to you in mental health policymaking?
   *Prompt:* Having influence, shared decision-making power, and co-leadership.
6. What support would make your participation in policy-making easier?
   *Probes:* transportation, training, compensation, and accessible meeting spaces.

**Closing**

Thank you all for participating and sharing your experiences and ideas. Your perspectives are essential to improving mental health policies and ensuring that lived experience voices are meaningfully included.
Before we finish, is there anything else anyone would like to add?
