# Supplementary material for: A Study Protocol: Engagement of Lived Experience Voices for Analysis of Transformative Evidence in Mental Health Policy & Legislation (ELEVATE-MH)
Source: PLoS One. 2026 Apr 15;21(4):e0346037. doi: 10.1371/journal.pone.0346037 (PMC13082620; doi:10.1371/journal.pone.0346037)
Supplement: S3 File — Participant demographics questionnaire administered to all study participants. (DOCX) [file pone.0346037.s004.docx]

**Appendix 4: Demographics Questionnaire**

1. What is your date of Birth?/ What is your age? mm/dd/yyyy
2. What is your gender? Male, Female, Other X
3. What country do you currently live in? Drop down menu of African countries (if someone answers a different country from Kenya, Malawi, South Africa, Ethiopia, and Liberia - If no, exclude participation)
4. How long have you lived in your country of residence? (0-6 months, 1- 5 years, more than 5 years)
5. What region do you live in? (Break down into counties/provinces)
6. What is your primary language? Secondary Language?
7. What is your nationality? (Type in their country of nationality)
8. What is the highest level of education that you have completed? No formal education, Some primary school, Completed primary School, Some high school, Completed high school, Some college, Completed college/Bachelor’s degree, Masters, Professional or Doctoral Degree.
9. What is your marital status? (single, living with a partner, married, separated , divorced

,widowed)

1. Do you belong to a faith community? No, African indigenous religion, Christianity, Islam, Other
2. What is your employment status? First option (employed, self employed, unemployed,) Options for Employed (Government, NGO, FBO, CSO, For-profit Org.) Options for
3. Self-Employed (Micro Ent.(Fewer than 10 employees) Small (Fewer than 50 Employees) Medium sized (up to 250 Employees) and Larger sized ( More than 250 employees)
4. Do you have Medical Insurance? (Yes, No, I do not know) If yes, what type? (Public, Private, both)
5. Does your Medical Insurance cover mental health concerns?(Yes, No, I do not know)

**Branching Question - Are you completing this as a Person With Lived Experience or a Policy Maker**

1. Do you consider yourself to have a disability? ( Yes, No, Prefer not to answer)
2. If you answered yes to question 14, please select the type of disability. *(Please select all that apply.)* Hearing difficulty, Learning disability/difficulty

(dyslexia, dyspraxia, dyscalculia, sensory processing disorder and others)n Mental health condition, Physical health condition, Neurodiversity (attention deficit hyperactivity disorder, autism and others),Vision impairment, Other *(please describe)*:

1. Have you ever been diagnosed with any of the following illnesses? (Check all that apply to you): Depression, Anxiety, ADHD, Psychosis, Bipolar 1, Bipolar 2, PTSD, Substance Use Disorder, Other
2. What is the duration of your illness? (less than 6 months, 1 year, 2 years, 3 years, 4 years, more than 5 years, more than 10 years
3. Are you receiving any interventions for your mental health diagnosis? (yes/no). If yes, what interventions (medication, psychotherapy, peer support, combination of 2 or more interventions etc)
4. Have you been prescribed psychiatric medication?
5. Are you currently taking psychiatric medication? If yes, how long have you been taking psychiatric medication?
6. Have you ever been diagnosed with any of the following? (Check all that apply to you): Asthma, Type 1 Diabetes, Type 2 Diabetes, Heart Disease, Kidney disease, Cancer, Lung disease: for example Emphysema or COPD (Chronic Obstructive pulmonary disease), Hypertension. Immunocompromised state (weakened immune system) from a solid organ transplant, Obesity, Serious heart conditions (heart failure, coronary artery disease or cardiomyopathies), Sickle cell disease.
7. Have you ever been diagnosed with any of the following illnesses? (Check all that apply to you): Depression, Anxiety, ADHD, Psychosis, Bipolar 1, Bipolar 2, PTSD, Substance Use Disorder, Other
8. What is the duration of your illness? (less than 6 months, 1 year, 2 years, 3 years, 4 years, more than 5 years, more than 10 years
9. Are you receiving any interventions for your mental health diagnosis? (yes/no). If yes, what interventions (medication, psychotherapy, peer support, combination of 2 or more interventions etc)
10. Have you been prescribed psychiatric medication?
11. Are you currently taking psychiatric medication? If yes, how long have you been taking psychiatric medication?
12. Have you ever been diagnosed with any of the following? (Check all that apply to you): Asthma, Type 1 Diabetes, Type 2 Diabetes, Heart Disease, Kidney disease, Cancer, Lung disease: for example Emphysema or COPD (Chronic Obstructive pulmonary disease), Hypertension. Immunocompromised state (weakened immune system) from a solid organ transplant, Obesity, Serious heart conditions (heart failure, coronary artery disease or cardiomyopathies), Sickle cell disease.
